# A Comparison of Fruit Chemical Characteristics of Two Wild Grown *Rubus* Species from Different Locations of Croatia

**DOI:** 10.3390/molecules170910390

**Published:** 2012-08-30

**Authors:** Dujmović Dubravka Purgar, Boris Duralija, Sandra Voća, Aleš Vokurka, Sezai Ercisli

**Affiliations:** 1 Faculty of Agriculture, University of Zagreb, Svetošimunska 25, 10000 Zagreb, Croatia; 2 Faculty of Agriculture, Ataturk University, 25240 Erzurum, Turkey

**Keywords:** *Rubus*, blackberry, raspberry, biodiversity, chemical properties

## Abstract

The main focus of our study was to investigate differences in nutritional (dry matter, soluble solids content, total acidity and pH value) and bioactive values (ascorbic acid, total anthocyanins, total phenols, and non-flavonoids content) of wild grown raspberry (*Rubus idaeus*) and blackberry (*Rubus discolor*) genotypes harvested from native populations in Croatia. The average total acidity ranged from 0.93 to 1.72% in *R. discolor* and 1.57 to 1.91% in *R. idaeus*. Ascorbic acid was found between 22.34 mg and 45.00 mg 100 g^−1^ in *R. idaeus*, while it was between 30.64 mg and 33.09 mg 100 g^−1^ in *R. discolor* genotypes. A great variability in total anthocyanins was detected in Croatian wild blackberry and raspberry genotypes, ranging from 2,226 to 2,367 mg kg^−1 ^for blackberries and 279 to 582 mg kg^−1^ for raspberries, indicating wild blackberries are particularly rich in anthocyanins. On the basis of these results, it can be concluded that investigated wild growing fruit species have a great potential in nutritive research, as well as in biodiversity research. It is necessary to carry out further investigation and evaluation of wild growing fruit species to utilize them in the most appropriate way, as well as conservation of interesting accessions in the gene banks.

## 1. Introduction

The Republic of Croatia is, despite its relatively small area (56,538 km^2^), one of the richest European countries in terms of plant diversity, due to its specific geographic position in South-East Europe and characteristic ecological, climatic and geomorphologic conditions. The country has mostly cultivated fruits along with wild forms of these. *Rubus* are one of the most abundant wild growing fruits in Croatia. Approximately half of all *Rubus* taxa found in Europe have also been found in different regions of the Republic of Croatia. According to available literature, 33 taxa within the genus *Rubus* are spread in Croatia [1]. Wild grown *Rubus* species are mostly found in forest edges, and numerous forests exist over wide areas of mountain and lowland in Croatia. Besides in forest edges, many members of wild grown genus *Rubus* could be found in vineyards, extensive orchards, hedges, pastures as well as in neglected meadows and other ruderal habitats [2]. Owing to long-lasting natural selection processes, wild *Rubus* species have adapted to the ecological conditions of their habitats and developed natural resistance mechanisms to biotic and abiotic environmental factors.

Small fruits including blackberries and raspberries are a good source of natural antioxidant substances and act effectively as free radical inhibitors [3]. They also contain high levels of phenolic compounds such as flavanols and anthocyanins [4], which contribute significant antioxidant and anticancer activity [5] and reduce the incidence of degenerative disease such as cancer and cardiovascular disorders [6]. The phenol, anthocyanin and ascorbic acid content of small fruits have been investigated [3,4,5,6,7,8,9,10,11,12,13,14,15]. It has been already demonstrated that a wide diversity of phytochemical levels exist within and across small fruit genera [6]. These berries were also found to be a rich source of bioactive compounds with considerable dieto-therapeutic impact on human health [5] and therefore interest in small fruits is increasing throughout the World. 

Plant breeding programmes have resulted with many fruit varieties being introduced in commercial production, but all these varieties have an origin in wild relatives. Cultivated varieties are extensively analysed and explored; on the other hand wild growing fruits species are rarely the subject of research. Therefore, detailed research with the objective of constructing a base inventory of the existing wild edible fruits genetic resources is needed, alongside with conservation of interesting accessions in the gene banks.

There seems to be a lack of information about the nutritional and bioactive content of wild grown small fruits including berries grown in different countries. Previous studies mostly addressed the basic chemical and overall pomological characteristics of wild berries. These studies mainly included blackberry and raspberry cultivars [3,4,5,6,8,9,10,11,12,16,17,18] because of an increased awareness of their possible health benefits as they are rich sources of micronutrients and phytochemicals such as organic acids, sugars and phenolics. The purpose of this research was to determine nutritional and bioactive content of wild grown berries in Croatia.

## 2. Results and Discussion

### 2.1. Basic Nutritional Properties

The basic nutritional composition of the wild berries is presented in the Table 1. There were statistically significant differences among genotypes in both species on most searched nutritional parameters, except pH (*p* < 0.01) (Table 1). We found dry matter content between 14.11 and 15.97% in *R. discolor* genotypes and 14.19–18.03% in *R. idaeus* genotypes (Table 1). Obtained results for dry matter content in *R. discolor* are in accordance with previously finding of Šoškić [17] who demonstrated a similar value for wild blackberry (17.36%). Our dry matter content for wild raspberries (*R. idaeus*) is higher than the value reported by Nalbandi *et al*. [15] for Iranian raspberry genotypes (14.02%).

**Table 1:** The basic nutritional properties in the fruit of wild berries (*R. discolor* and *R. idaeus*).

Species	Sample	Dry matter content (%)	Total acidity (%)	pH	Total soluble solids content (^0^Brix)
*Rubus discolour* (blackberry)	1	15.17 ± 1.1 ab	1.34 ± 0.2 b	3.14 ± 0.3 ^NS^	5.10 ± 0.5 c
	2	15.97 ± 1.2 a	1.72 ± 0.3 a	3.24 ± 0.2	7.40 ± 0.3 a
	3	14.11 ± 0.8 b	0.93 ± 0.1 c	3.45 ± 0.2	6.80 ± 0.2 b
*Rubus idaeus* (raspberry)	1	14.19 ± 1.3 d	1.89 ± 0.1 a	3.16 ± 0.2 ^NS^	10.88 ± 0.6 ab
	2	18.03 ± 1.1 a	1.57 ± 0.2 b	3.18 ± 0.3	11.50 ± 0.7 a
	3	17.25 ± 1.4 b	1.59 ± 0.1 b	3.16 ± 0.2	10.20 ± 0.6 ab
	4	15.45 ± 1.2 c	1.91 ± 0.2 a	3.06 ± 0.1	9.40 ± 0.4 b

Means within a column followed by the same letter are not significantly different at *p* = 0.01; NS: Non significant.

Total acidity of *R. discolor* in our research ranged from 0.93 to 1.72%. Previously total acidity content in registered blackberry cultivars was in the range of 1.09 to 1.30% [17], indicating agreement with our study. Total acidity content for wild raspberries varied from 1.57 to 1.91%. Fotirić *et al*. [16] reported total acidity between 0.55 and 1.14% for selected raspberry genotypes.

In the present study, the pH ranged from 3.06 to 3.18 in *R. idaeus* and 3.14 to 3.45 in *R. discolor*. Our results of pH in *R. discolor* are within the range of the values reported by Šoškić [17] and Yilmaz *et al*. [11]. Šoškić [17] reported pH values between 3.20 and 4.38 in blackberries. The pH result showed that native Croatian raspberries are comparable to Iranian raspberries [15].

The content of total soluble solids ranged from 5.10 to 7.40 ^0^Brix in *R. discolor* to 9.40–11.50 ^0^Brix in *R. idaeus*, showing that there were great variability among the examined berry fruits. Total soluble solids content in *R. discolor* (5.10–6.80 ^0^Brix) were lower than values reported by other authors [6,11,17]. In contrast, the total soluble solids in blackberry cultivars range from 10 to 12% [17] and 9.8 to 11.5% [6]. The total soluble solids of *R. idaeus* in our research were within the range of 9.4–11.5 ^0^Brix. It is higher compared to raspberry cultivars (7.1–10.8%) [6] or Iranian raspberry genotypes (7%) [15], but within the range of 9.95 to 12.80% [16] seen in Serbia.

The most nutritional properties indicate that the species *R. discolor* have a lower levels of dry matter, total soluble solids and total acidity, but pH was higher in some samples. *R. idaeus* ranked higher for all basic nutritional compounds, except for pH, which was lower, then in *R. discolor*.

### 2.2. Bioactive Contents

Important bioactive contents of examined fruit of wild berries (*R. discolor* and *R. idaeus*) are shown in the Table 2. As indicated in Table 2, mostly significant differences were seen among parameters.

**Table 2:** The nutritional properties in the fruit of wild berries (*R. discolor* and *R. idaeus*).

Species	Sample	Ascorbic acid (vit C) content	Total anthocyanin content	Total phenol Content	Nonflavonoids content
		(mg/100gFW)	(mg/kgFW)	(mg/kg FW)	(mg/kgFW)
*Rubus discolor* (blackberry)	1	33.09 ± 2.2 ^NS^	2358 ± 21 ^NS^	585 ± 10 a	292 ± 6 b
	2	32.90 ± 1.9	2226 ± 20	481 ± 8 b	289 ± 6 b
	3	30.64 ± 1.8	2367 ± 21	381 ± 7 c	315 ± 7 a
*Rubus idaeus* (raspberry)	1	45.00 ± 2.4 a	470 ± 9b	377 ± 9 bc	226 ± 6 ab
	2	33.19 ± 1.9 b	582 ± 11 a	483 ± 9 a	246 ± 7 a
	3	22.34 ± 1.5 c	341 ± 9 c	354 ± 8 c	204 ± 5 b
	4	30.09 ± 1.7 b	279 ± 8 d	396 ± 7 b	208 ± 5 ab

Means within a column followed by the same letter are not significantly different at *p* = 0.01; NS: Non significant.

There are significant differences in ascorbic acid content among native blackberries in Croatia. Ascorbic acid value of *R. discolor* genotypes varied from 30.64 to 33.09 mg 100 g^−1^ and were between 22.34 and 45.00 in *R. idaeus* genotypes (Table 2). These results reveal that ascorbic acid levels are more variable in *R. idaeus*. As well known ascorbic acid influences the antioxidant value of berry fruits. Contrary to this study, Pantelidis *et al*. [6] recorded a lower ascorbic acid content of Greek blackberry cultivars, which was within the range of 14.3–17.5 mg 100 g^−1^. Voća *et al*. [18] reported ascorbic acid as 14.50 mg 100 g^−1^ for *Thornless logan* and 17.42 mg 100 g^−1^ for *Black Satin* fruits. 

According to the literature, raspberry cultivars contain approximately 25.00 mg 100 g^−1^ ascorbic acid [5], or 27.50 mg 100 g^−1^ ascorbic acid [10]. Our data (22.34–45.00 mg 100 g^−1^ ascorbic acid) are similar to those reported by Milivojević *et al*. [12] for wild raspberry. On the other hand, ascorbic acid was not detected in some wild ecotypes in Turkey [13]. Milivojević *et al.* [12] determined the uniform values for ascorbic acid contained in cultivated raspberries ranging from 40.9 mg 100 g^−1^ FW (Willamette) to 44.3 mg 100 g^−1^ FW (Meeker), whereas somewhat higher value was recorded for *R. idaeus* harvested from native populations of Serbia (56.1 mg 100 g^−1^ FW). In other studies, the ascorbic acid content of raspberry cultivars from Greece was within the range 31 and 40 mg 100 g^−1^ [6], which was followed by raspberry cultivars from Norway with 27.5 mg 100 g^−1^ [10]. Considering the results obtained for ascorbic acid content in this study, only *R. discolor* fruits exhibited higher levels compared to commercial blackberry cultivars grown in Croatia. On the other hand, the similar levels of ascorbic acid as registered in cultivars characterized the native raspberry. The ascorbic acid content from this study is comparable to the previously reported results obtained for wild berries and cultivars [18].

We found great differences in anthocyanins within and between blackberries and raspberries (Table 2) and blackberries were found to be the richer. They have approximately 7-8 fold higher anthocyanins than raspberries (Table 2). In the present study, *R. discolor* demonstrated the most abundant quantity of anthocyanins in sample 3 (2367 mg kg^−1^), and this value was almost 2-fold higher compared to the data obtained for cultivars Thornless logan and Thorn free published by Voća *et al*. [18]. Similar differences between native and cultivar blackberries were reported by Milivojević *et al*. [12]. A great variation of total anthocyanins was detected in Croatian wild raspberry ranging from 279 to 582 mg kg^−1^. This has already been reported by previous researchers who found 3- to 4-fold higher values for native raspberry [12].

Anthocyanins are the predominant group of flavonoids present in berries, being the pigments that are responsible for the red and blue colour of these fruits. According McGhie *et al*. [9], the anthocyanin content in the *Rubus* fruits varied considerably, with the lowest level in red raspberry and the highest in blackberry [5]. 

Total phenol content of native blackberry (*R. discolor*) genotypes in our research was within range 382–586 mg kg^−1^ (Table 2). Among native blackberries (*R. discolor*) investigated in this study, the highest amounts of total phenolics were registered in genotype 1 (586 mg kg^−1^). Comparing to results of native blackberries from Croatia and Turkey, there is a large difference between them. Total phenolic content of wild materials from Turkey [11] was between 610 mg and 1,455 mg 100 g^−1^ fresh weight. Milivojević *et al*. [12] reported total phenol content of blackberry cultivar within the range 1.74–1.97 mg g^−1^, and 3.20 mg g^−1^ in the native blackberry. The native blackberry total phenolic content indicate higher value then total phenolic content in cultivars [18], which was within the range 338–376 mg kg^−1^ fresh weight. Wild blackberry germplasm had a higher total phenolic content than cultivated blackberries. This phenomenon is explained in Yilmaz *et al*. [11] as an induction in the synthesis of antioxidant enzymes and an increase in polyphenolic concentration brought about due to the greater exposure of the unsheltered wild plants to extremes of temperature, and insult by pests and disease organisms, because phenolic compound synthesis is typically a defensive mechanism.

The total phenolic content differed greatly among the native raspberries tested in this research achieving the highest value in genotype 2 (483 mg kg^−1^). Milivojević *et al*. [12] reported the total phenol content for raspberry cultivars within the range 1.02–2.22 mg g^−1^, and 1.10 mg g^−1^ for native raspberry. Recently, Gülçin *et al*. [13] in Turkey determined phenolic contents of wild raspberries that varied from 5.83 mg GAE to 26.66 mg GAE mg extract. Previously, Pantelidis *et al*. [6] in Greece determined phenolic contents of raspberries cultivars that varied from 657 to 2494 mg kg^−1^ of GAE. Total phenolic content of raspberry cultivars was within range 359–512 mg 100 g^−1^ [5].

Phenolic compounds contribute to fruit quality and nutritional value in terms of modifying colour, taste, aroma, and flavour [11]. Phenolic compounds in a diet rich in fruits and vegetables have attracted the attention of researchers due to their health promoting attributes, which include lowering the risk of cardiovascular disease, cancer or other condition with the ageing process [4]. Particularly, phenols contribute substantially to the antioxidant component of many small fruit species, having potential health effects [6]. 

Non-flavonoids content ranged from 204 to 246 mg kg^−^^1^ in *R. idaeus* and 289 to 315 mg kg^−^^1^ in *R. discolor* (Table 2). This results indicating that there were significant differences among genotypes belongs to blackberry and raspberry.

All these bioactive substance values indicates that the *R. discolor* species have a higher content of total anthocyanins, total phenols and non-flavonoids then *R. idaeus*. The significant factors that may influence the difference in values obtained for native blackberry or raspberry are the laboratory techniques employed, as well as whether the data were collected from whole-fruits or only fruit juice. The genetic background, the degree of ripeness, climatic factors and postharvest storage also contribute to the differences.

Our study may have provides valuable information on the basic nutritional properties (dry matter, total acidity, pH and total soluble solids) and bioactive properties (ascorbic acid, total anthocyanin content, total phenol content, non-flavonoids) of wild blackberry and raspberry species grown in Croatia. Also, this information should prove valuable for similar climatic areas in other parts of the World.

## 3. Experimental

### 3.1. Study Area

The study area is located in the northwest of Croatia, in the Medvednica Mountain area, ranging from 230 to 1,000 meters above sea level (m a.s.l., Table 3). The climate is continental middle European type, middle warm and rainy climate with warm summer without dry period. The mean annual temperature ranges from 5.5 to 12.7 °C, the coldest months are January or December (−7.6–6.5 °C) and the warmest are July and August (13.6–25 °C). Precipitation ranges from 821.4 mm to 1,228.4 mm/year, most of which falls in late autumn; a less pronounced secondary peak occurs in late spring.

**Table 3:** Sampling location of the wild berries (*R. discolor* and *R. idaeus*) used in this study.

Species	Sample	Longitude	Latitude	Altitude
*Rubus discolour* (blackberry)	1	N 45°55'27.81''	E 15°58'29.80''	686 m a.s.l.
	2	N 45°51'21.99''	E 16°00'17.25''	233 m a.s.l.
	3	N 45°55'30.17''	E 15°58'34.85''	666 m a.s.l.
*Rubus idaeus* (raspberry)	1	N 45°52'49.86''	E 15°58'13.27''	529 m a.s.l.
	2	N 45°54'15.05''	E 15°58'08.63''	971 m a.s.l.
	3	N 45°53'25.29''	E 15°56'08.44''	966 m a.s.l.
	4	N 45°53'01.56''	E 15°56'28.94''	635 m a.s.l.

### 3.2. Plant Material Collection and Extraction

Collections were carried out in July and September 2007 in the area of Medvednica Mountain including the Medvednica Nature Park. Seven types of *Rubus* spp. were determined previously [19]. The most prevalent species were *Rubus discolor* and *Rubus idaeus*. Spots of individual species of *R. discolor* and *R. idaeus* were determined with a GPS device (Garmin GPS 60).

The *Rubus discolor* population was distributed in three different locations, whereas the *Rubus idaeus* were found in four different locations within the Medvednica Mountain (Table 3). The fruit samples were collected from these locations for further analyses in the laboratory of food technology at the Faculty of Agriculture, University of Zagreb, Croatia.

Fruit sample were extracted and analyzed immediately after harvesting. The fruits were mashed in laboratory homogeniser (Mixy, Zepter International, New York, NY, USA) and marked for further analysis. Three replicates were used per analysis.

### 3.3. Determination of Basic Nutritional Properties

The following basic nutritional parameters of harvested fruits were determined: dry matter (total and soluble), total acids (TA), pH value and ascorbic acid (vitamin C).

Total dry matter (DM) was obtained by drying homogenised berries at 105 °C until constant mass [20]. Total soluble solids (TSS), expressed in ºBrix, were measured using an Abbe refractometer (A. Krüss Optronic, Hamburg, Germany) calibrated against sucrose. Total acidity (TA) was measured according to the AOAC method [20] and expressed in g/L citric acid. PH value was measured with a pH meter (Mettler Toledo, Greifensee, Switzerland). Ascorbic acid (AA) was determined by the 2.6-dichloroindophenol titrimetric method according to the AOAC method [20].

### 3.4. Determination of Bioactive Content

The anthocyanin content in the extract from selected fruits was determined using the bisulphite bleaching method [21]. Fruit anthocyanins were extracted from fresh samples (2 g) using 0.1% HCl (by volume, 2 mL) in 96% ethanol and 2% aqueous HCl (by volume, 40 mL). The mixture was centrifuged at 5,500 rpm for 10 min. The obtained supernatant was used for the determination of total anthocyanin. The content of total anthocyanin was measured as follows: extract (10 mL) was put into two test tubes, then 15% sodium bisulphite (4 mL) was added to one tube and ddH_2_O (4 mL) to the other. After 15 min of incubation at room temperature, the absorbance of each mixture was measured at 520 nm. The molar absorbance value for cyanidin-3,5-diglucoside was used as a standard value. Results were expressed as mg of cyanidin-3,5-diglucoside equivalents/kg of fresh mass of edible part of fruits.

Total phenolics (TP) and non-flavonoids (TNF) were determined using the Folin-Ciocalteu colorimetric method described by Ough and Amerine [22] with some modifications. Fruit phenolics were extracted from fresh samples (10 g) using 80% (by volume) aqueous ethanol (40 mL). The mixture was extracted (in water bath at 80 °C), kept for 20 min in inert atmosphere, and filtered through a Whatman filter paper using a Büchner funnel. Extraction of the residue was repeated under the same conditions. The filtrates were combined and diluted to 100 mL in volumetric flask with 80% aqueous ethanol, and the obtained extract was used for determination of TP and TNF. The content of TNF was measured as follows: 0.5 mL of diluted extract or standard solutions of gallic acid (20–500 mg/L) was added to a 50-mL volumetric flask containing ddH_2_O (30 mL), then Folin-Ciocalteu reagent (2.5 mL) was added to the mixture and shaken. After 5 min, 7% Na_2_CO_3_ solution (7.5 mL) was added with mixing and the solution was immediately diluted to 50 mL with ddH_2_O. After incubation at room temperature for 2 h the solution was measured by using spectrophotometer (Perkin Elmer, Waltham, MA, USA) at 760 nm absorbance. TP and TNF were expressed as mg of gallic acid equivalents (GAE)/kg of fresh mass of edible part of fruits.

### 3.5. Statistical Analysis

All data were analyzed using SAS software and procedures (Chicago, IL, USA). Analysis of variance tables were constructed using the Least Significant Difference (LSD) method at *p* < 0.01.

## 4. Conclusions

No studies on nutritional and bioactive characteristics of native raspberry and blackberry have been conducted so far in the Republic of Croatia. Our study may have provides valuable information on the basic nutritional properties (dry matter, total acidity, pH and total soluble solids) and bioactive properties (ascorbic acid, total anthocyanin content, total phenol content, non-flavonoids) of wild blackberry and raspberry species grown in Croatia. Also, this information should be valuable for similar areas all over the World. It can be concluded that the investigated wild growing fruit species have a great future potential in nutritive research, as well as in biodiversity research. It is necessary to carry out further inventorisation and evaluation of investigated wild growing fruit species to utilize them in the most appropriate way, as well as for conservation of interesting accessions in the gene banks.
